# Study on postoperative survival prediction model for non-small cell lung cancer: application of radiomics technology workflow based on multi-organ imaging features and various machine learning algorithms

**DOI:** 10.3389/fmed.2025.1517765

**Published:** 2025-02-05

**Authors:** Hanlin Wang, Yuan Hong, Zimo Zhang, Kang Cheng, Bo Chen, Renquan Zhang

**Affiliations:** ^1^Department of Thoracic Surgery, The First Affiliated Hospital of Anhui Medical University, Hefei, China; ^2^Department of General Surgery, The First Affiliated Hospital of Anhui Medical University, Hefei, China; ^3^Department of The First Clinical Medical College, Anhui Medical University, Hefei, China

**Keywords:** NSCLC, erector spinae muscle, radiomics, artificial intelligence, prognosis

## Abstract

**Objective:**

This study aims to construct an effective prediction model for the two-year postoperative survival probability of patients with non-small cell lung cancer (NSCLC). It particularly focuses on integrating radiomics features, including the erector spinae and whole-lung imaging features, to enhance the accuracy and stability of prognostic predictions.

**Materials and methods:**

The study included 37 NSCLC patients diagnosed and surgically treated at the First Affiliated Hospital of Anhui Medical University from January 2020 to December 2021. The average age of the patients was 59 years, with the majority being female and non-smokers. Additionally, CT imaging data from 98 patients were obtained from The Cancer Imaging Archive (TCIA) public database. All imaging data were derived from preoperative chest CT scans and standardized using 3D Slicer software. The study extracted radiomic features from the tumor, whole lung, and erector spinae muscles of the patients and applied 11 machine learning algorithms to construct prediction models. Subsequently, the classification performance of all constructed models was compared to select the optimal prediction model.

**Results:**

Univariate Cox regression analysis showed no significant correlation between the collected clinical factors and patient survival time. In the external validation set, the K-Nearest Neighbors (KNN) model based on bilateral erector spinae features performed the best, with accuracy and AUC (Area Under the Curve) values consistently above 0.7 in both the training and external testing sets. Among the prognostic models based on whole-lung imaging features, the AdaBoost model also performed well, but its AUC value was below 0.6 in the external validation set, indicating overall classification performance still inferior to the KNN model based on erector spinae features.

**Conclusion:**

This study is the first to introduce erector spinae imaging features into lung cancer research, successfully developing a stable and well-performing prediction model for the postoperative survival of NSCLC patients. The research results provide new perspectives and directions for the application of radiomics in cancer research and emphasize the importance of incorporating multi-organ imaging features to improve the accuracy and stability of prediction models.

## 1 Introduction

Lung adenocarcinoma, the major subtype of non-small cell lung cancer (NSCLC), often presents with nonspecific symptoms such as persistent cough, hemoptysis, chest pain, and dyspnea. These symptoms frequently lead to delayed diagnosis, as they are easily attributed to less severe respiratory conditions. Advanced imaging techniques and histopathological examinations are crucial for accurate diagnosis. The overall prognosis for lung adenocarcinoma patients is generally poor, particularly in advanced stages, with survival rates significantly influenced by the stage at diagnosis, molecular characteristics, and treatment response. Early-stage lung adenocarcinoma may have a better prognosis, with a five-year survival rate of 60–70% if surgical resection is possible. However, for advanced and metastatic stages, the prognosis remains grim, with median survival times usually measured in months. Emerging targeted therapies and immunotherapies show promise in improving outcomes for specific genetic subgroups, emphasizing the importance of personalized medicine in lung adenocarcinoma treatment strategies (1, 2).

Sarcopenia, characterized by the progressive loss of muscle mass and strength, is common among older adults and significantly impacts their quality of life. Clinically, sarcopenia manifests as muscle weakness, reduced stamina, and difficulty performing daily activities (3). The condition is associated with adverse outcomes, including increased risk of falls, fractures, physical disability, and mortality (4). Prognosis varies depending on severity and the presence of comorbidities, but early intervention through resistance exercises and adequate protein intake can improve outcomes. Regular physical activity and nutritional support are crucial in managing sarcopenia and preventing its progression (5). Loss of muscle mass is an important sign of aging, thus making sarcopenia a hot topic in geriatric research (6). As research deepens, the relationship between sarcopenia and tumor prognosis has gradually become another research hotspot, especially in gastric and esophageal cancers (7, 8). However, in the field of oncology, sarcopenia is not clearly defined. Cancer-related sarcopenia studies rely on CT-determined muscle mass as a diagnostic criterion. More complexly, various CT-derived cut points have been used to characterize normal muscle mass in cancer patients. The definitions of sarcopenia in gerontological and oncological literature partially differ due to different perspectives and outcomes considered (9). This paper explores the relationship between sarcopenia and lung cancer prognosis by studying the erector spinae muscles.

In the past decade, with the rapid development of artificial intelligence (AI), more researchers have been using AI techniques to process medical images. In fact, computer algorithms have been used in radiology since the 1960s. In recent years, this field has remained a research hotspot, particularly in the study of tumor images (10). AI has revolutionized medical imaging, enhancing diagnostic accuracy and efficiency. AI algorithms, especially deep learning models, can analyze complex imaging data, such as X-rays, CT scans, and MRIs, to identify disease indicators with remarkable precision. For example, AI systems can detect early signs of conditions like lung cancer, stroke, and diabetic retinopathy, often with accuracy comparable to or surpassing that of experienced radiologists (11). Additionally, AI helps quantify disease progression, segment anatomical structures, and automate routine tasks, allowing clinicians to focus on more critical decision-making aspects. Integrating AI into medical imaging not only improves diagnostic outcomes but also facilitates personalized treatment plans, ultimately advancing the field of precision medicine (12).

Although some studies have explored the relationship between sarcopenia and lung cancer, most of these studies have focused on assessing the impact of general muscle mass (such as abdominal muscle measurements using CT scans), with sample sizes often limited to patients with advanced lung cancer (13, 14). There is limited research on specific muscles in the thoracic region, particularly the erector spinae muscles. The importance of the erector spinae in lung cancer patients cannot be ignored (15). These muscles not only play a crucial role in maintaining posture, supporting the spine, and assisting with respiration but are also among the major muscle groups in the thoracic region. Therefore, changes in the muscle mass of the erector spinae may significantly influence the prognosis of lung cancer.

In this study, we constructed NSCLC prognostic models using tumor imaging features, lung imaging features, and erector spinae imaging features, and compared their performance. By analyzing CT images, we used 11 machine learning algorithms and integrated imaging data from multiple organs to construct a highly accurate prognostic prediction model for lung cancer patients. The motivation behind this study is to use imaging technology to precisely assess the muscle mass of the erector spinae and explore its clinical significance in lung cancer patients (16–18). We aim to specifically investigate the relationship between the erector spinae and lung cancer prognosis, evaluating the impact of sarcopenia on key clinical outcomes such as survival time, treatment response, and postoperative recovery. Through this study, we hope to provide a new, muscle-based prognostic marker for clinical use. Specifically, in the context of imaging visualization and quantitative analysis, the erector spinae may serve as a more sensitive and specific prognostic tool.

## 2 Materials and methods

### 2.1 Clinical cohort and data collection

**Figure 1:**
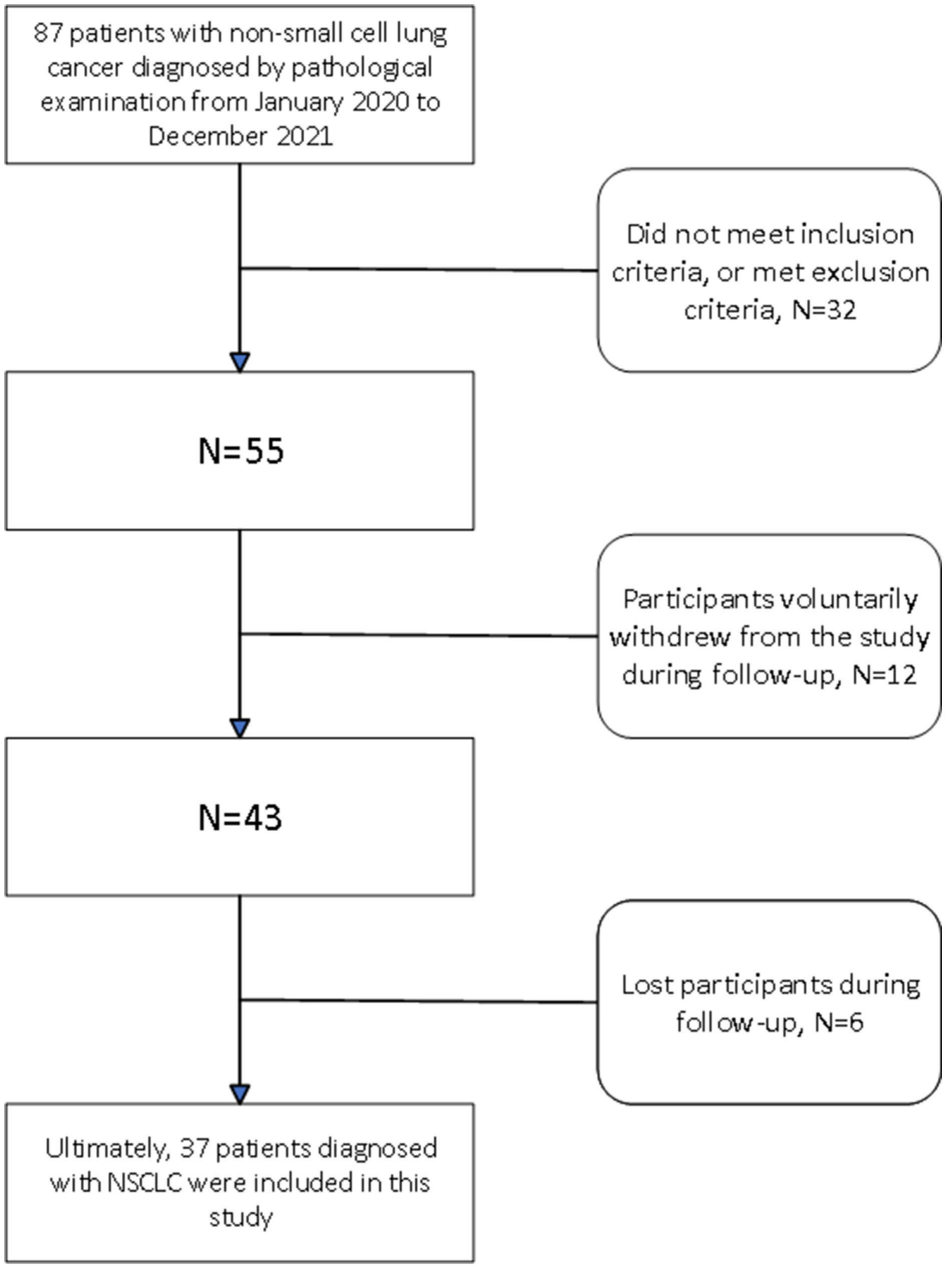
Clinical cohort establishment flowchart.

This study received formal approval from the Ethics Committee of the First Affiliated Hospital of Anhui Medical University. The retrospective study focused on the period from January 2020 to December 2021, documenting detailed clinical records of 55 pathologically confirmed NSCLC patients at our hospital. The inclusion and exclusion criteria are detailed in Supplementary material 1. During the study period, this patient group did not suffer from other cancers, nor did they have severe fatal complications such as organ failure or cardiovascular events. All patients met the surgical criteria and successfully underwent radical lung cancer surgery at our hospital after CT scans. The surgical procedures included thoracoscopic wedge resection, segmentectomy, and lobectomy. Following surgery, our hospital implemented continuous follow-up according to standard protocols. By June 2024, 12 patients had withdrawn from the study due to personal reasons, and 6 were lost to follow-up, resulting in missing prognostic data. Currently, we have successfully collected two-year survival data for the remaining 37 patients, with survival time recorded monthly for future data analysis and research applications (shown in Figure 1).

Additionally, the research team retrieved two datasets from the TCIA public database that met the study requirements, completing the download and integration (19–21). The links to the two datasets are provided later. Patients were excluded from the study based on several criteria to ensure the quality and relevance of the data. Cases with incomplete or low-quality CT images, which were deemed insufficient for radiomics analysis, were excluded. Additionally, patients lacking essential clinical metadata—such as age, sex, diagnostic information, and survival data (1-year and 2-year)—were not considered. Patients with diagnoses other than lung adenocarcinoma, including squamous cell lung cancer and small cell lung cancer, were also excluded. Furthermore, cases affected by artifacts, noise, or incomplete regions of interest (ROIs) that would hinder accurate segmentation and feature extraction were excluded from the analysis. This public dataset includes preoperative chest CT scan information and more than 2 years of prognostic data, providing 98 additional valid cases for this study. Thus, the total dataset for this study covers 135 NSCLC patient cases from three centers.

### 2.2 Clinical cohort statistical analysis

The data collected from 37 patients at our center included 11 clinical indicators, such as height, weight, and TNM staging, which were comprehensively analyzed by the research team (details in Table 1). To ensure patient confidentiality, all clinical data were anonymized. During statistical analysis, the team used SPSS 27.0 software for rigorous data processing and GraphPad Prism software for precise graphical representation.

**Table 1:** Characteristics of the clinical population.

Variable		*N* = 37
Age(years)		59.65 ± 1.5
Gender	Male	12 (0.32)
	Female	25 (0.68)
Body height(cm)		164.27 ± 1
Body weight(kg)		62.42 ± 1.5
BMI		23.09 ± 0.46
Smoking status	Non-smoking	32 (0.86)
	Smoking	5 (0.14)
Number of lymph nodes removed		12.24 ± 1.02
Number of lymph nodes invaded		0.78 ± 0.33
T-stage	Tis	1 (0.03)
	T1	21 (0.57)
	T2	14 (0.38)
	T3	1 (0.03)
N-stage	N0	28 (0.76)
	N1	6 (0.16)
	N2	2 (0.05)
	N3	1 (0.03)
M-stage	M0	37 (1)
Vascular cancer thrombus	Positive	9 (0.24)
	Negative	28 (0.76)
Neurological invasion	Positive	1 (0.03)
	Negative	36 (0.97)
Pleural invasion	Positive	10 (0.27)
	Negative	27 (0.73)
Vacuolar sign	Positive	6 (0.16)
	Negative	31 (0.84)
Calcification	Positive	1 (0.03)
	Negative	36 (0.97)
Two-year survival rate	Dead	4 (0.11)
	Alive	33 (0.89)

For statistical methods, the chi-square test was employed to assess differences in categorical variables, while normality tests were first conducted for continuous variables, followed by the appropriate t-test or Mann–Whitney U test to ensure precise results. Numerical variables were presented as medians (with interquartile ranges) and means ± standard deviations.

In the field of survival analysis, the team initially utilized univariate Cox regression analysis to preliminarily screen for variables potentially related to survival time. To further uncover the complex relationships among factors, highly relevant clinical data were included in a multivariate Cox regression model for in-depth identification and modeling, ultimately determining the factors significantly impacting survival time. Survival analysis results are detailed in Table 2.

**Table 2:** The results of cox univariate analysis.

Variables	*β*	S.E	Z	*p*	HR (95%CI)
Age(years)	0.03	0.06	0.52	0.603	1.03 (0.92 ~ 1.16)
Body height(cm)	0.05	0.08	0.65	0.514	1.05 (0.90 ~ 1.23)
Body weight(kg)	−0.06	0.07	−0.78	0.435	0.94 (0.82 ~ 1.09)
BMI	−0.37	0.28	−1.31	0.190	0.69 (0.40 ~ 1.20)
Lymph	−0.13	0.11	−1.12	0.262	0.88 (0.71 ~ 1.10)
Lymph nodes invaded	0.38	0.22	1.69	0.091	1.46 (0.94 ~ 2.25)
Gender					
Male					1.00 (Reference)
Female	−0.36	1.15	−0.32	0.752	0.69 (0.07 ~ 6.68)
Smoking status					
Non-smoking					1.00 (Reference)
Smoking	0.76	1.15	0.66	0.512	2.13 (0.22 ~ 20.51)
T					
Tis					1.00 (Reference)
T1	18.36	25659.08	0.00	0.999	94055544.02 (0.00 ~ Inf)
T2	17.67	25659.08	0.00	0.999	47027772.01 (0.00 ~ Inf)
T3	0.00	36287.42	0.00	1.000	1.00 (0.00 ~ Inf)
N					
N0					1.00 (Reference)
N1	2.23	1.22	1.82	0.068	9.33 (0.85 ~ 102.93)
N2	−15.47	12116.31	−0.00	0.999	0.00 (0.00 ~ Inf)
N3	2.64	1.41	1.87	0.062	14.00 (0.88 ~ 223.83)
Vascular cancer thrombus					
Positive					1.00 (Reference)
Negative	1.13	1.00	1.13	0.256	3.11 (0.44 ~ 22.09)
Neurological invasion					
Positive					1.00 (Reference)
Negative	−17.04	15068.02	−0.00	0.999	0.00 (0.00 ~ Inf)
Pleural invasion					
Positive					1.00 (Reference)
Negative	−0.11	1.15	−0.09	0.927	0.90 (0.09 ~ 8.65)
Vacuolar sign					
Positive					1.00 (Reference)
Negative	−19.28	17501.47	−0.00	0.999	0.00 (0.00 ~ Inf)
Calcification					
Positive					1.00 (Reference)
Negative	−17.04	15068.02	−0.00	0.999	0.00 (0.00 ~ Inf)

HR, Hazard Ratio; CI, Confidence Interval.

Notably, due to patient confidentiality principles, the two public databases did not provide detailed clinical information for each patient, posing a limitation for this study.

### 2.3 Radiomics workflow

**Figure 2:**
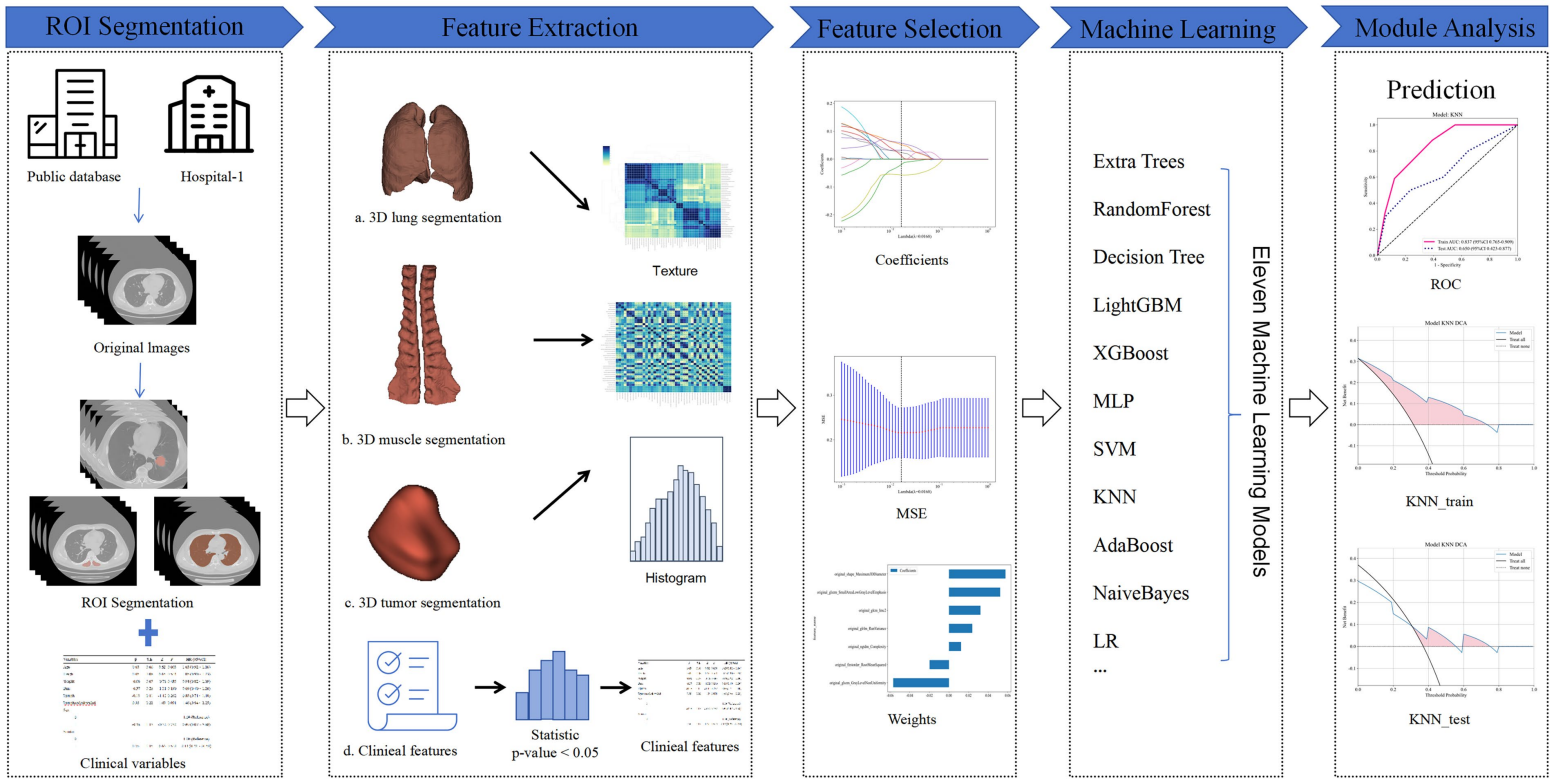
Radiomics workflow diagram.

This study employed a radiomics workflow to construct prognostic models, including data acquisition, ROI delineation, feature extraction, and AI model construction. The specific workflow is illustrated in Figure 2. Our image segmentation process was designed to target biologically relevant regions, including tumors, lungs, and erector spinae muscles, all of which hold prognostic value in lung cancer studies. Using the TotalSegmentator v2 auto-segmentation tool, we ensured efficient segmentation, complemented by radiologist supervision to maintain clinical accuracy. For feature extraction, we applied a Fixed Bin Number discretization strategy to address the heterogeneity of lung cancer data and employed Spearman correlation and redundancy removal techniques to minimize overfitting. Lasso regression was used for feature selection, ensuring the inclusion of statistically and clinically significant variables. To evaluate model robustness, we tested 11 machine learning algorithms across both public (e.g., TCIA) and private datasets, using ROC and DCA curves to identify high-performance models with clinical interpretability. Our study prioritizes the application of AI-driven medical imaging to solve clinical challenges, with a focus on prognostic outcomes rather than delving into the scientific theories behind each radiomics step. However, scientifically sound methodologies were incorporated at each stage to address the specific challenges of lung cancer prognosis.

#### 2.3.1 Acquisition and standardization of imaging data

The research team obtained public imaging data from the TCIA, consisting of chest CT scan images with original settings retained. The public database data had been meticulously annotated with tumor target regions and accompanied by patient prognostic information. The CT dataset from our research center was derived from preoperative chest CT scans of patients. Image acquisition was performed using a 256-channel Philips Brilliance i CT scanner (Philips, Eindhoven, Netherlands). During the scans, a 512 × 512 matrix was used to ensure in-plane resolution remained at a high level of 0.62 × 0.62 mm to 0.86 × 0.86 mm, ensuring high image clarity. Radiomics workflow quality assessment for the acquired data are detailed in Supplementary checklist 1 (22) and Supplementary checklist 2 (23).

To ensure consistency between our research center’s imaging data and the public database, the research team used 3D Slicer software to set the CT image window width (WW) to 149 and window level (WL) to 40, adjusting the threshold range to −35 to 115. Additionally, to ensure multi-center imaging data consistency, the research team performed resampling on all imaging data, standardizing voxel size to 3 mm × 3 mm × 3 mm.

#### 2.3.2 ROI delineation

**Figure 3:**
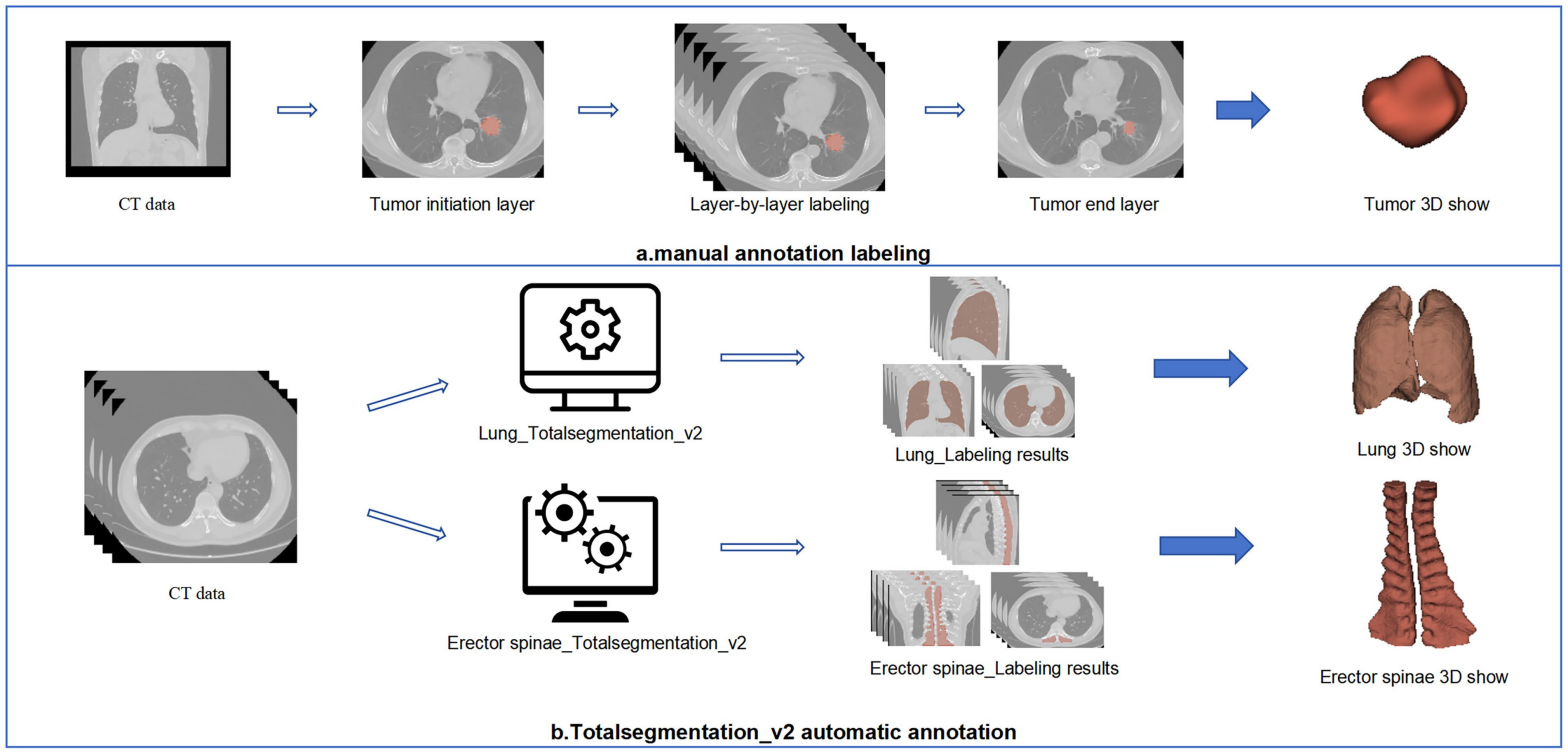
Multiple organ sketching flowchart.

Figure 3 presents the complete ROI delineation workflow. The study required all lung cancer patients to delineate three target areas: the tumor, the entire lung, and bilateral erector spinae muscles. During the delineation process, we used the TotalSegmentation v2 auto-delineation tool to efficiently delineate the lungs and bilateral erector spinae muscles. The complete code and scripts are publicly available on GitHub. Since the tool was not developed by us, we did not provide a detailed description in the paper to avoid any confusion regarding the attribution of the code. If you are interested, you can refer to the following link: TotalSegmentator V2 GitHub repository. The radiologists involved in the image segmentation process have an average of 7 years of experience in imaging and have received specialized training in tumor segmentation. Three senior radiologists are certified by our center. During the image segmentation process, the three radiologists reached a consensus through discussion. Although we did not formally report the consistency metrics, the disagreements were minimal, and all issues were resolved by consensus. Additionally, the file format was converted from DICOM to nii.gz before delineation to meet subsequent processing and analysis requirements.

#### 2.3.3 Feature extraction and engineering

We selected 5 as the bin number parameter for the Fixed Bin Number (FBN) discretization technique (24). This choice was based on both literature recommendations and preliminary experiments, which demonstrated that five bins provided the most uniform feature distribution and optimal statistical stability. The FBN method was chosen to ensure consistent feature extraction across heterogeneous datasets. During preprocessing, we used the sitkNearestNeighbor interpolation method, which preserved critical edge details required for texture and shape-based radiomic analysis. To further standardize the images, resampling filters were applied to align voxel spacing across datasets, minimizing the impact of imaging protocol variability (25, 26).

After completing the feature extraction step, we proceeded with the regularization of features. This step is crucial as it scales or standardizes the feature values, making different features comparable and facilitating subsequent data processing and analysis. To comprehensively evaluate the correlation between features, we used statistical T-tests and calculated the Spearman correlation coefficient. The Spearman correlation coefficient is a non-parametric measure that assesses the monotonic relationship between two variables, suitable for data not meeting the normal distribution assumption. When the correlation coefficient between feature pairs exceeded 0.9, indicating high correlation, we implemented redundancy removal measures. Specifically, we retained only one feature from each highly correlated pair to simplify the model structure, avoid overfitting, and enhance the model’s generalization ability.

**Figure 4:**
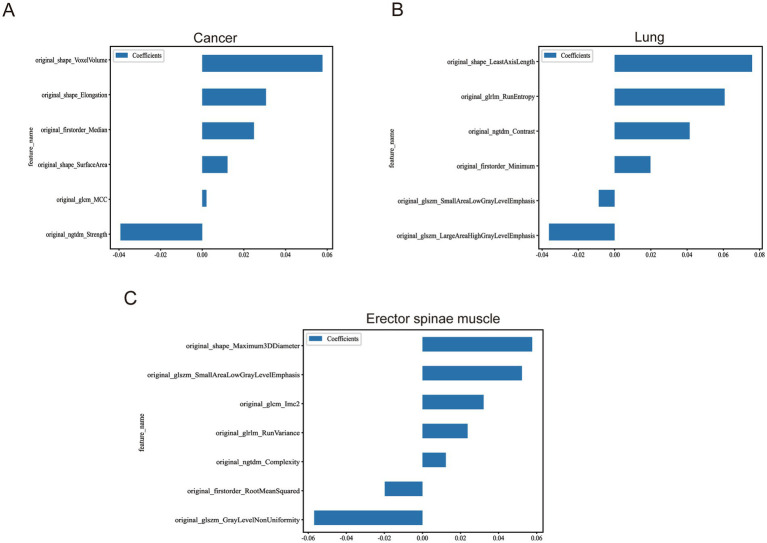
Statistics of image features after multi-organ screening. **(A)** Remaining image features after screening of tumour images. **(B)** Remaining image features after screening of lung images. **(C)** Remaining image features after screening of the erector spinae images.

Building on this, we further initialized the Lasso (Least Absolute Shrinkage and Selection Operator) regression model. Lasso regression introduces an L1 regularization term, allowing for the automatic selection of features that significantly contribute to the predictive target (i.e., non-zero regression coefficients). This step is vital for constructing a streamlined and efficient prediction model. Detailed information on the engineering processes is provided in Table 3, which outlines the operational key points and parameter settings for each step. Meanwhile, the extracted feature statistics, including feature value distribution and correlation analysis, are visually presented in Figure 4.

**Table 3:** Radiomics feature extraction settings.

Setting	Determination
Bin Method	FBN
Bin Amount	5
Method	SitkNearestNeighbor
Resample Filter	1
Resample Spacing X	3 mm
Resample Spacing Y	3 mm
Resample Spacing Z	3 mm

FBN, fixed bin number.

#### 2.3.4 Modeling using machine learning algorithms

In constructing the prediction models, we carefully selected key features to serve as inputs for various machine learning algorithms. To efficiently train and validate the models, the research team first performed a scientific split of the dataset, which included data from 98 patients in the public database, into training and testing sets at an 8:2 ratio. This step ensured that the models could adequately learn the intrinsic patterns in the data during training and validate their performance in the subsequent internal testing set.

After identifying the optimal prediction model in the training and internal testing sets, we further applied this model to the dataset of 37 patients collected at our center to observe and evaluate the model’s generalization performance. This step is crucial for verifying the model’s generalizability and practical applicability.

Regarding model selection, the research team conducted extensive and meticulous screening work. We comprehensively considered multiple factors, including algorithm performance, stability, and interpretability, ultimately selecting 11 machine learning algorithms for modeling experiments. These algorithms, each with unique characteristics, include mainstream and cutting-edge machine learning techniques such as SVM (Support Vector Machine), KNN (K-Nearest Neighbor), RandomForest, ExtraTrees, XGBoost (eXtreme Gradient Boosting), LightGBM (Light Gradient Boosting Machine), NaiveBayes, AdaBoost (Adaptive Boosting), GradientBoosting, LR (Logistic Regression), and MLP (Multi-Layer Perceptron) (27–30).

After obtaining the model prediction results, we employed various methods to evaluate and compare the models’ performance. Firstly, by plotting ROC curves and calculating accuracy, we could intuitively understand the model’s ability to distinguish between different sample categories. Additionally, to analyze the impact of classification thresholds on prediction results more deeply, we also plotted DCA (Decision Curve Analysis) curves.

## 3 Results

### 3.1 Analysis of clinical factors in patients

The research team systematically summarized 17 clinical data points from the patients, details of which are clearly presented in Table 1. These data are carefully divided into two categories: categorical data and continuous variables. Specifically, the categorical data include 11 items: gender, smoking habits (whether the patient smokes), TNM staging, presence of vascular invasion, neural invasion, pleural involvement, presence of cavitation, calcification, and the survival status of patients within 2 years. These categorical data are detailed in terms of their numbers and proportions for an intuitive understanding of the distribution of each category. On the other hand, continuous variables include the patients’ age, height, weight, body mass index (BMI), the number of lymph nodes removed during surgery, and the number of metastatic lymph nodes, totaling six items. For continuous variables, we used the mean and standard deviation, a common statistical method, to describe the data, aiming to accurately reflect data central tendency and dispersion.

To explore which clinical factors could serve as independent factors affecting patients’ postoperative survival, the research team used a Cox proportional hazards regression model for survival analysis, with the patients’ two-year postoperative survival time as the dependent variable. Unfortunately, the analysis results showed that the collected clinical factors did not show a significant correlation with the patients’ survival prognosis, with specific analysis results referenced in Table 2. Additionally, it is particularly noteworthy that all patients included in this study were in the M0 stage, meaning they had no distant metastasis, which is a basic prerequisite for lung cancer surgery treatment. Therefore, M staging clinical data were not included in the survival analysis to avoid result interference.

### 3.2 Interpretation of radiomics results

#### 3.2.1 Tumor radiomics

In exploring the construction of prognostic prediction models for NSCLC patients using tumor imaging data, we encountered a series of challenges that resulted in the overall model performance not meeting expectations. The accuracy of the models and detailed data are shown in Table 4. Specifically, we attempted to apply 10 mainstream machine learning algorithms, including NaiveBayes, SVM, KNN, RandomForest, ExtraTrees, XGBoost, LightGBM, GradientBoosting, AdaBoost, and MLP. However, when these models were tested on the 37 external validation sets meticulously collected by our center, their performance was significantly poor, with accuracy not reaching the statistically acceptable threshold of 0.5. This result clearly indicates that these models may lack sufficient robustness and generalization ability in practical clinical classification tasks.

**Table 4:** The prediction results of each model based on tumor imaging features.

Model_name	Task	Accuracy	AUC
LR	training	0.577	0.644
	Internal val	0.75	0.76
	External val	0.514	0.523
NaiveBayes	training	0.603	0.661
	Internal val	0.85	0.76
	External val	0.459	0.591
SVM	training	0.308	0.19
	Internal val	0.75	0.48
	External val	0.297	0.447
KNN	training	0.769	0.832
	Internal val	0.4	0.627
	External val	0.351	0.5
RandomForest	training	0.936	0.964
	Internal val	0.7	0.64
	External val	0.216	0.383
ExtraTrees	training	0.897	0.959
	Internal val	0.75	0.667
	External val	0.297	0.379
XGBoost	training	0.872	0.942
	Internal val	0.45	0.62
	External val	0.432	0.538
LightGBM	training	0.782	0.764
	Internal val	0.8	0.807
	External val	0.486	0.466
GradientBoosting	training	0.949	0.978
	Internal val	0.7	0.56
	External val	0.459	0.466
AdaBoost	training	0.744	0.871
	Internal val	0.7	0.693
	External val	0.486	0.489
MLP	training	0.603	0.689
	Internal val	0.8	0.72
	External val	0.486	0.5

**Figure 5:**
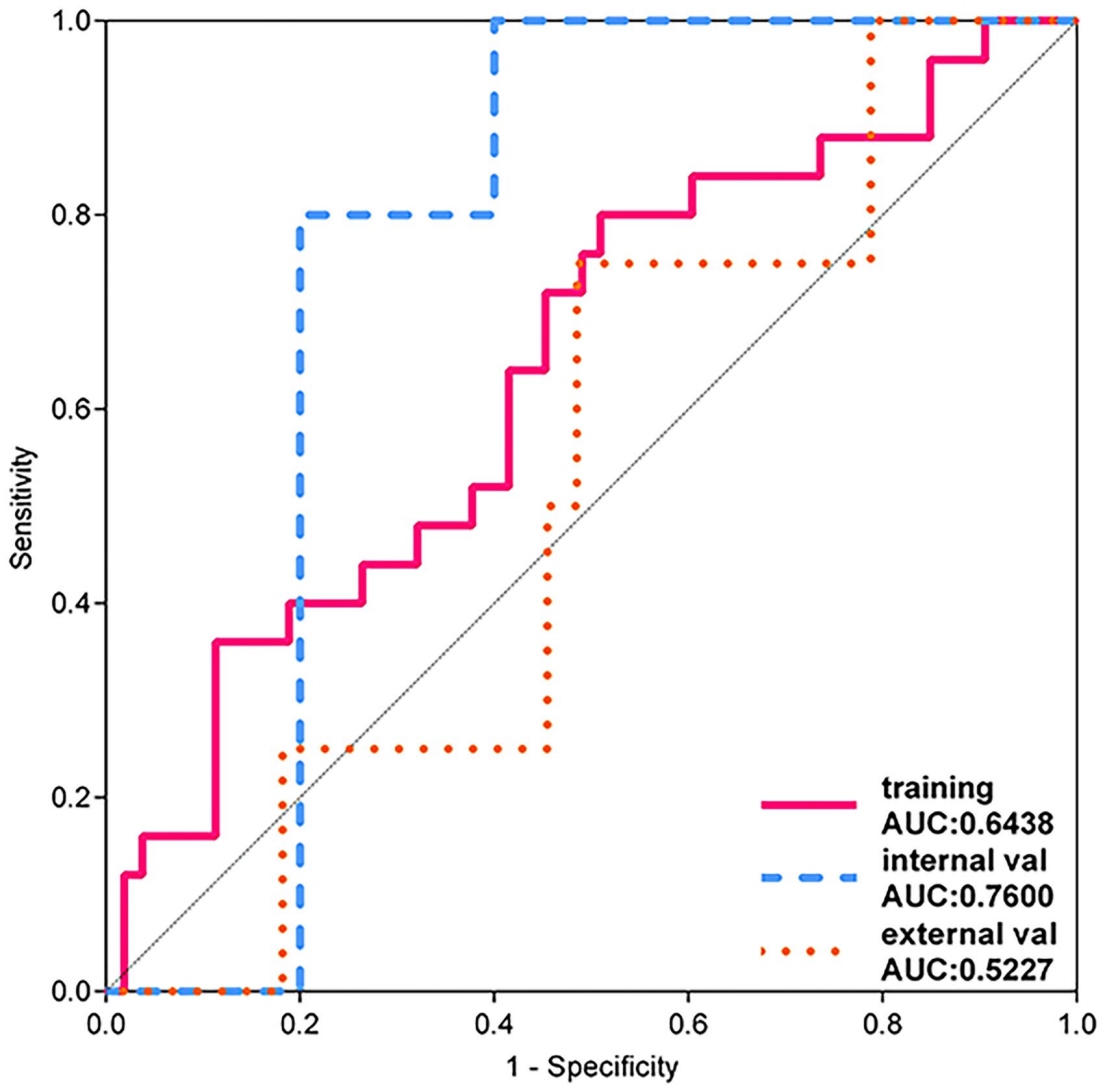
LR model ROC curve.

Notably, although the LR model barely achieved an accuracy above 0.5 on the external validation set, its performance was unstable (see Figure 5). Specifically, the confidence intervals of AUC for the LR model in the training set, internal validation set, and external validation set showed considerable dispersion, indicating that the model’s predictive performance may vary significantly across different datasets. Therefore, despite the LR model potentially demonstrating some predictive ability in certain cases, we still find it challenging to assert that it possesses robust and reliable diagnostic performance.

#### 3.2.2 Lung imaging radiomics

In exploring the use of automated delineation technology to introduce whole-lung imaging features into machine learning models to predict expected survival, we evaluated 11 different algorithms. Results indicated improvements in predictive performance compared to the baseline. However, in-depth analysis revealed significant performance differences between models, with detailed data shown in Table 5.

**Table 5:** The prediction results of each model based on lung imaging features.

Model_name	Task	Accuracy	AUC
LR	training	0.654	0.672
	Internal val	0.85	0.893
	External val	0.568	0.523
NaiveBayes	training	0.679	0.676
	Internal val	0.85	0.92
	External val	0.568	0.538
SVM	training	0.705	0.682
	Internal val	0.65	0.627
	External val	0.568	0.576
KNN	training	0.705	0.73
	Internal val	0.55	0.727
	External val	0.784	0.576
RandomForest	training	0.885	0.965
	Internal val	0.8	0.827
	External val	0.486	0.553
ExtraTrees	training	0.808	0.906
	Internal val	0.85	0.853
	External val	0.622	0.511
XGBoost	training	0.897	0.929
	Internal val	0.7	0.747
	External val	0.622	0.587
LightGBM	training	0.667	0.762
	Internal val	0.9	0.907
	External val	0.622	0.542
GradientBoosting	training	0.936	0.951
	Internal val	0.75	0.853
	External val	0.865	0.538
AdaBoost	training	0.769	0.85
	Internal val	0.8	0.853
	External val	0.703	0.591
MLP	training	0.692	0.682
	Internal val	0.85	0.933
	External val	0.568	0.523

Specifically, the model derived from the RandomForest algorithm failed to achieve the 0.5 accuracy threshold in the external validation set, indicating insufficient predictive ability. Similarly, the models based on LR, NaiveBayes, MLP, and SVM, despite showing some potential, did not exceed a prediction accuracy of 0.6 in the external validation set, a level generally considered insufficient to demonstrate good classification performance. On the other hand, the XGBoost and GradientBoosting algorithms exhibited outstanding performance in the training set; however, their AUC values significantly declined in the external validation set, suggesting possible overfitting to the training set. Therefore, these models were not considered optimal choices. Additionally, the LightGBM model showed signs of overfitting in the internal validation set.

**Figure 6:**
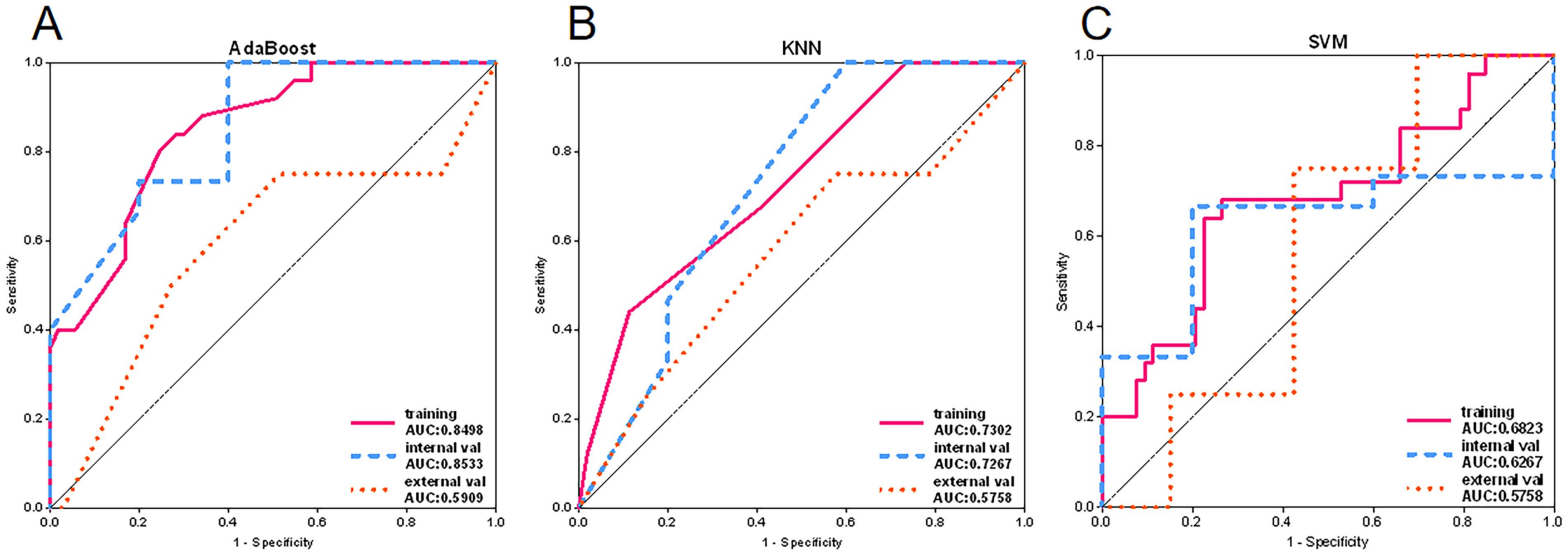
Adaboost, KNN and SVM model ROC curves. **(A)** ROC curves of Adaboost model in training set, internal validation set and external validation set. **(B)** ROC curves of KNN model in training set, internal validation set and external validation set. **(C)** ROC curves of SVM models in training set, internal validation set and external validation set.

Among the remaining models, the AdaBoost model’s classification performance stood out (see Figure 6). In the external validation set, its AUC value was closest to 0.6, and its prediction accuracy was similar to the best results during training, indicating that the model not only had good internal consistency but also demonstrated some extrapolation ability. Therefore, the AdaBoost model was considered the optimal choice in this study.

#### 3.2.3 Erector spinae radiomics

In the process of constructing predictive models and incorporating erector spinae features, we observed a significant increase in AUC values for the series of models in the external validation set. This result suggests that the models might possess excellent generalization performance, with specific data detailed in Table 6. Further in-depth analysis of model performance revealed that the NaiveBayes, LR, XGBoost, and AdaBoost algorithms did not reach the 0.5 accuracy threshold in the external validation set. This led us to conclude that these models lacked efficacy in practical classification applications. Moreover, from the perspective of classification efficiency, the GradientBoosting, LightGBM, SVM, and MLP models also failed to reach the 0.6 accuracy threshold in the external validation set, indicating unsatisfactory performance in classification tasks.

**Table 6:** The prediction results of each model based on erector spinae muscle imaging features.

Model_name	Task	Accuracy	AUC
LR	training	0.705	0.737
	Internal val	0.7	0.56
	External val	0.486	0.689
NaiveBayes	training	0.654	0.775
	Internal val	0.8	0.573
	External val	0.378	0.523
SVM	training	0.731	0.72
	Internal val	0.75	0.48
	External val	0.568	0.705
KNN	training	0.731	0.889
	Internal val	0.6	0.713
	External val	0.838	0.72
RandomForest	training	0.949	0.983
	Internal val	0.65	0.573
	External val	0.676	0.761
ExtraTrees	training	0.872	0.929
	Internal val	0.75	0.62
	External val	0.622	0.648
XGBoost	training	0.885	0.943
	Internal val	0.7	0.567
	External val	0.378	0.659
LightGBM	training	0.744	0.805
	Internal val	0.6	0.513
	External val	0.541	0.727
GradientBoosting	training	0.936	0.972
	Internal val	0.7	0.58
	External val	0.568	0.723
AdaBoost	training	0.821	0.948
	Internal val	0.6	0.633
	External val	0.351	0.515
MLP	training	0.705	0.801
	Internal val	0.75	0.6
	External val	0.514	0.659

**Figure 7:**
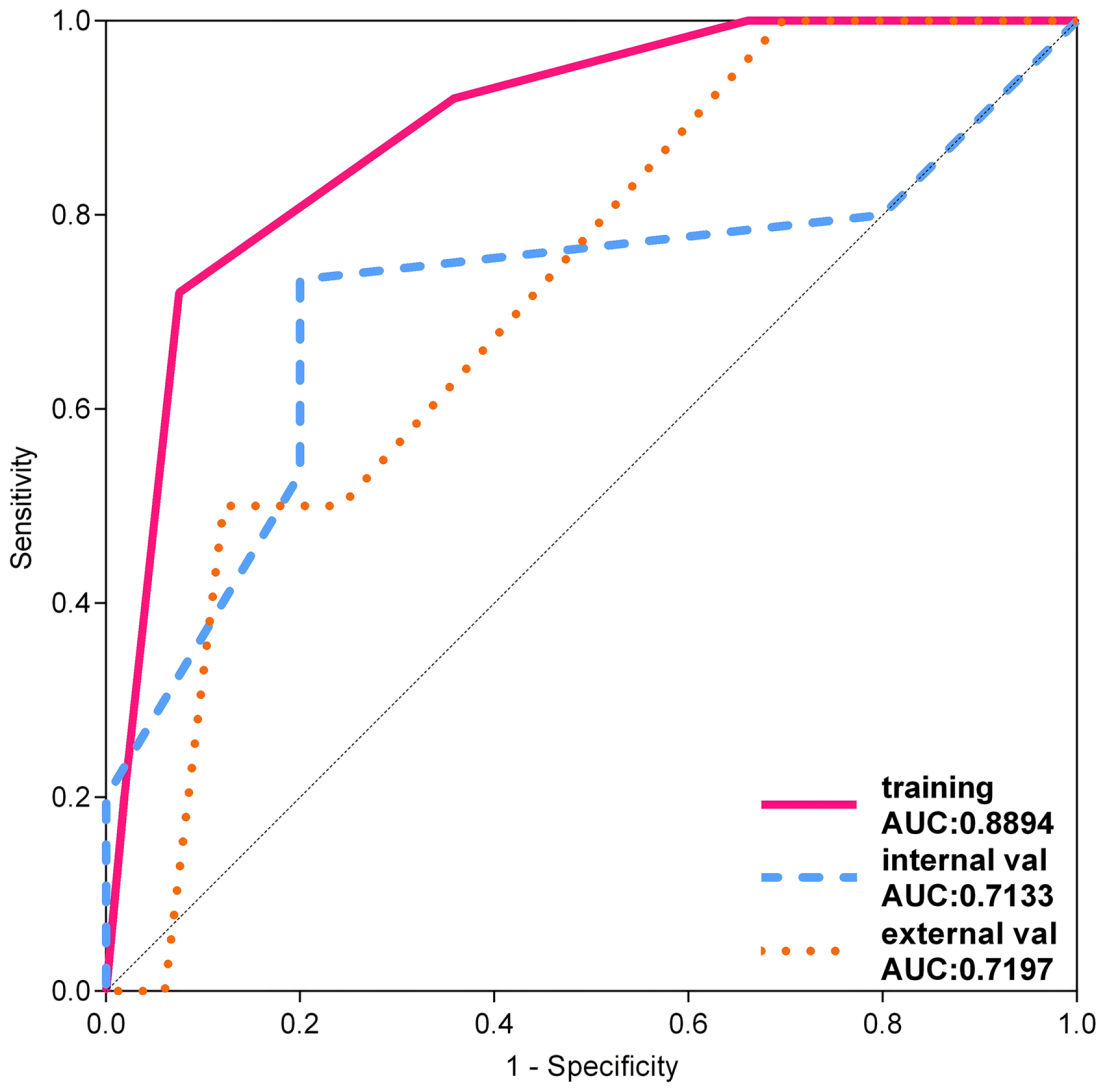
ROC curve of KNN model.

Among the remaining models, the RandomForest model showed significant overfitting in the training set, challenging its reliability in practical applications. Additionally, the notable difference in AUC values between the training and testing sets for the ExtraTrees model raised concerns about the risk of overfitting. However, the KNN model maintained stable accuracy and AUC values in both the training and testing sets, validating its robustness and generalizability, as shown by the ROC curve in Figure 7.

#### 3.2.4 Comparison of optimal models

**Figure 8:**
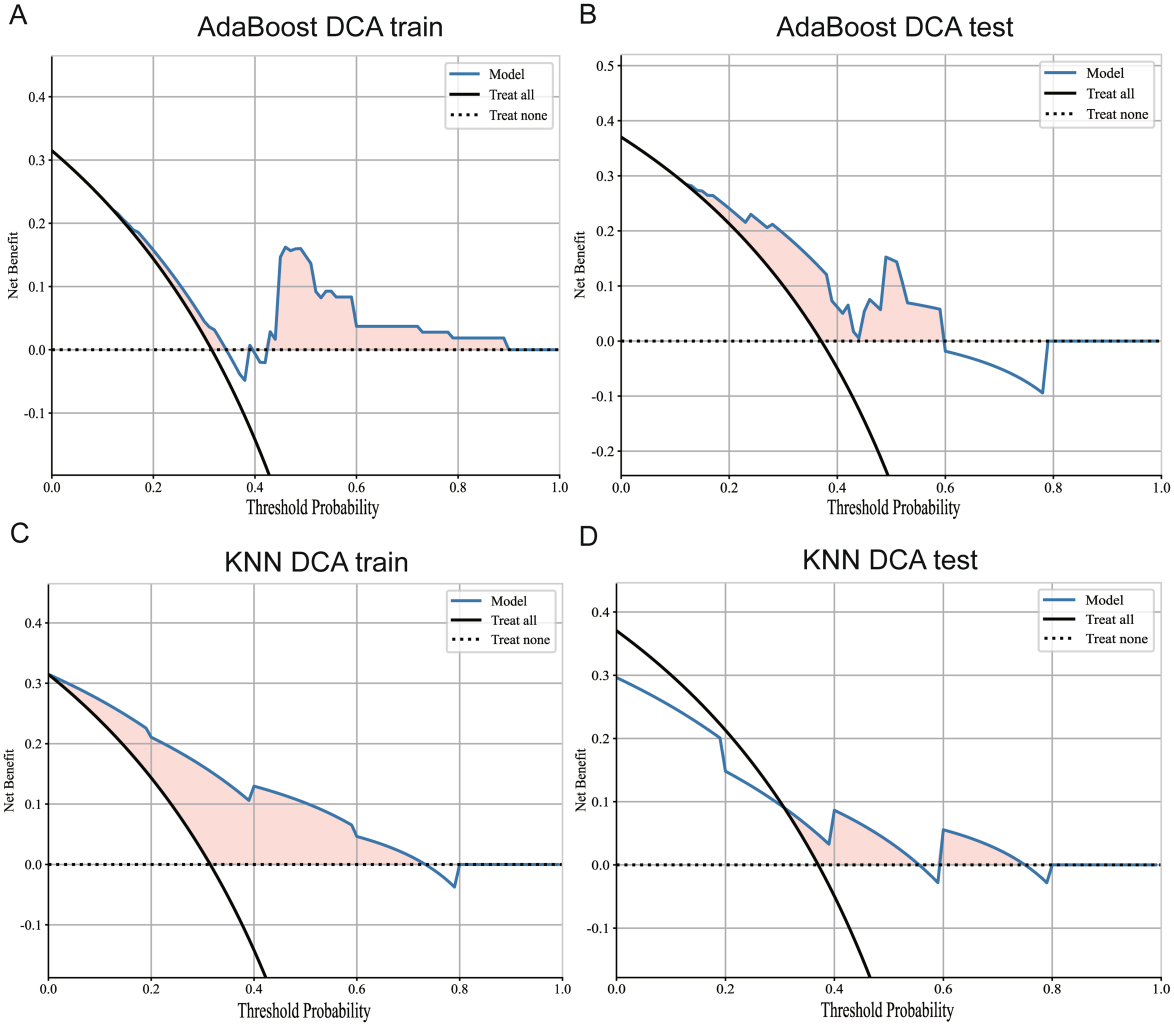
DCA curves for Adaboost and KNN models. **(A)** DCA curve of Adaboost model in training set. **(B)** DCA curve of Adaboost model in external validation set. **(C)** DCA curve of KNN model in the training set. **(D)** DCA curves for KNN models in external validation sets.

After a comprehensive analysis of 33 prognostic prediction models constructed using tumor, lung, and bilateral erector spinae imaging features, we reached several key conclusions. Specifically, models built using tumor imaging features demonstrated the lowest accuracy in the external validation set, as detailed in Figure 8. In contrast, prediction models constructed using lung imaging and bilateral erector spinae imaging features showed comparable accuracy in the external validation set.

To more comprehensively evaluate these models’ performance, we compiled the AUC values for each predictive model in the external validation set across the three modalities (tumor, lung, and erector spinae), with specific data provided in Table 7. Through comparative analysis, we found that predictive models built using erector spinae imaging features significantly improved AUC values in the external validation set. This discovery further prompted us to conduct a difference analysis, comparing the erector spinae modality with the other two modalities, and the results also confirmed the superiority of erector spinae imaging features in the predictive models.

**Table 7:** Model external validation set AUC comparison based on tumor, lung and erector spinae imaging features.

	LR	NaiveBayes	SVM	KNN	RandomForest	ExtraTrees	XGBoost	LightGBM	GradientBoosting	AdaBoost	MLP	mean	z	*p*
Erector spinae muscle imaging features	0.689	0.523	0.705	0.72	0.761	0.648	0.659	0.727	0.723	0.515	0.659	0.66 ± 0.08		
Tumor imaging features	0.523	0.591	0.447	0.5	0.383	0.379	0.538	0.466	0.466	0.489	0.5	0.48 ± 0.06	−3.62^a^	<0.001
Lung imaging features	0.523	0.538	0.576	0.576	0.553	0.511	0.587	0.542	0.538	0.591	0.523	0.55 ± 0.03	−2.73^a^	0.005

a Mann–Whitney method test was used.

Based on the above analysis, we conclude that the prognostic prediction model based on bilateral erector spinae features demonstrated the best performance in terms of predictive capabilities. The model’s accuracy and AUC values consistently remained above 0.7 in both the training and external testing sets. Furthermore, in the external validation set for diagnosing patient survival, the accuracy and AUC of this model were also higher than those of models based on lung imaging features. This result partially reveals the potential association between muscle mass and the survival period of lung cancer patients.

**Figure 9:**
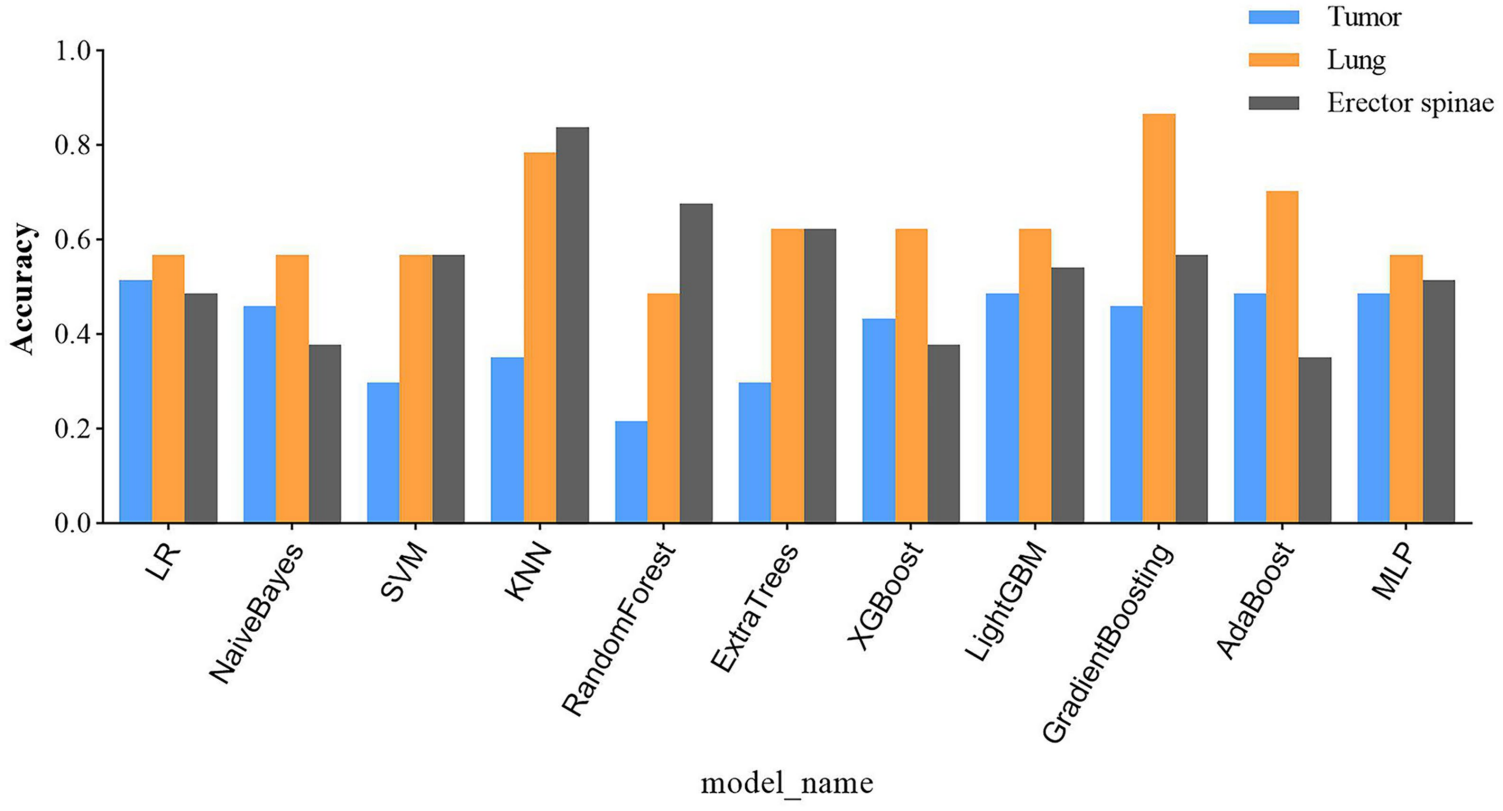
Prediction accuracy of all predictive models on external validation set.

Additionally, we conducted DCA of the best models for whole-lung radiomics and erector spinae radiomics, as shown in Figure 9. The results indicate that both models provided positive benefits to classification performance at a threshold level of 0.5. Therefore, within a selected threshold range, both models offer practical classification value. However, from the prediction results, the KNN model still demonstrated the best classification performance. In summary, this study not only verified the superiority of prognostic prediction models based on bilateral erector spinae features in predicting the survival period of lung cancer patients but also provided a new research direction for future exploration of cancer patient survival using muscle imaging.

## 4 Discussion

This study meticulously collected 17 clinical baseline data points from patients. Univariate Cox regression analysis showed no correlation between the collected clinical information and patients’ postoperative survival time. As a result, the research team did not create a survival prognosis model based on our center’s clinical data. Additionally, the patients from the TCIA database lacked personal clinical information, precluding further in-depth study. In our research, we used machine learning methods to construct a prognostic prediction model for lung cancer based on multi-organ imaging data. Among them, 37 pathologically confirmed NSCLC patients from our center were included, with an average age of 59 years, predominantly female and non-smokers. These patients underwent surgical treatment and lymph node dissection. Most were in the early stages of lung cancer (stage I). During the first- and second-years post-surgery, we conducted telephone follow-ups, with four patients having passed away (31). The research team also used tumor imaging data from the TCIA to construct a prognostic prediction model for NSCLC patients. During this process, we attempted to apply machine learning algorithms, including NaiveBayes, SVM, KNN, RandomForest, ExtraTrees, XGBoost, LightGBM, GradientBoosting, AdaBoost, LR, and MLP. However, when tested on our center’s imaging data, none of the models performed well and did not reach the statistically acceptable accuracy threshold of 0.5. We speculate this might be due to the early stage of the included NSCLC patients and the short follow-up period. Subsequently, we used automatic contouring techniques to introduce whole-lung imaging features into machine learning models to predict prognosis. Among these 11 models, the AdaBoost model showed outstanding classification performance. In the external validation set, its AUC value was closest to 0.6, and its prediction accuracy was similar to the best results during training, indicating that the model not only had good internal consistency but also showed some extrapolation ability. Other models performed poorly and did not reach the expected accuracy threshold. Therefore, in this study, the AdaBoost model was considered the best choice.

The prognosis of lung cancer patients is influenced by several key factors. Tumor staging and lymph node metastasis are critical determinants, the more advanced the stage and the higher the number of lymph node metastases, the poorer the prognosis. Patient age and overall health are also important, as older patients and those with comorbidities generally have worse outcomes (32, 33). With further research, sarcopenia has also been recognized as an important factor affecting lung cancer prognosis. In our study, the imaging features of the erector spinae muscles could predict the prognosis of NSCLC patients to some extent, which is consistent with previous studies.

Sarcopenia, characterized by the loss of muscle mass and function, is a significant prognostic factor in lung cancer patients (34). Studies have shown that sarcopenia is highly prevalent among lung cancer patients, with a prevalence ranging from 42.8 to 45.0%. The presence of sarcopenia is associated with poorer functional status, increased postoperative complications, and decreased overall survival. Thus, early evaluation of muscle mass and function is crucial (35, 36). We incorporated erector spinae features in constructing predictive models and observed a significant increase in AUC values in the external validation set. The KNN model maintained stable accuracy and AUC values in both the training and testing sets, validating its robustness and extrapolation. Due to low accuracy and overfitting, other models were discarded. In conclusion, the prognostic prediction model based on bilateral erector spinae features demonstrated the best performance. The model’s accuracy and AUC values remained above 0.7 in both the training and testing sets. Furthermore, compared to models based on lung imaging features, this model showed better classification performance in diagnosing patient survival, providing new perspectives and possibilities for AI applications in the medical field.

Radiomics, which extracts quantitative features from medical images, has made significant strides in lung cancer diagnosis and treatment. By analyzing imaging data from CT scans, PET scans, and MRIs, radiomics can identify subtle patterns associated with tumor characteristics, such as genetic mutations and histological subtypes. This method shows promise in improving the accuracy of lung cancer detection, predicting treatment responses, and personalizing therapy plans (37–39). In recent years, the development of AI has further propelled advances in medical imaging. Deep learning models, especially convolutional neural networks (CNNs), are now routinely used to analyze medical images, detecting subtle anomalies that might be overlooked by the human eye (40). These AI-driven systems excel at identifying early signs of diseases such as cancer, cardiovascular disorders, and neurological conditions, facilitating early interventions and improving patient outcomes. Additionally, AI algorithms can automate image segmentation, reducing the workload for radiologists and minimizing human error. Integrating AI into medical imaging workflows provides quantitative data and predictive analytics, promoting the development of personalized treatment plans (41). In this study, we constructed prognostic models for NSCLC patients using tumor imaging features, lung imaging features, and erector spinae imaging features, and conducted a thorough comparison using 11 machine learning algorithms. By analyzing CT images, we utilized machine learning techniques to construct a high-precision prognostic prediction model for NSCLC patients based on erector spinae imaging features.

One important finding of our study is that the degree of muscle depletion in the erector spinae is correlated with worse clinical outcomes. These results are in line with the findings of previous studies, who observed that sarcopenia was an independent predictor of poor prognosis in NSCLC patients (35, 42). However, unlike many previous studies, we included a broader range of lung cancer patients, not limiting our sample to those with advanced disease. This inclusion of early-stage patients adds depth to our analysis, suggesting that sarcopenia may play a role in prognosis even at earlier stages of lung cancer, which has not been as well documented in prior research. This study addresses several key gaps in the field of radiomics and medical imaging. It integrates multi-modal imaging data, combining information from lung cancer tumors, the entire lung, and bilateral erector spinae muscles, thus improving diagnostic accuracy and prognostic prediction. The research goes beyond typical radiomics studies by conducting a comprehensive analysis of shape, texture, and intensity features, offering a more nuanced understanding of imaging data. Validation across multiple datasets from diverse clinical settings ensures broader applicability and robustness of the models. Furthermore, the study emphasizes the clinical relevance of radiomics, translating findings into practical tools for real-time decision support and personalized patient care. By integrating CT and employing advanced machine learning techniques, the study enhances diagnostic accuracy for lung cancer, aiding early detection and improving outcomes. Additionally, prognostic models based on radiomics enable more precise risk stratification and treatment planning. Methodological innovations in feature selection and model validation establish a benchmark for future research. Ultimately, this study paves the way for the broader adoption of radiomics-based models in clinical settings, promising a transformative impact on patient management through personalized medicine.

Despite the strength of these findings, several limitations must be considered. First, in this study, only traditional machine learning methods were used, not deep learning methods. Secondly, our study relied on automated segmentation tools for muscle mass quantification, which may introduce variability depending on the accuracy of the segmentation algorithms. Although we performed quality control checks and resolved discrepancies through consensus among the radiologists, more research into the precision and reliability of these automated methods is necessary to validate their use in clinical settings. Thirdly, the number of patients included from our center is relatively small. Finally, we did not perform multimodal fusion to build potentially more accurate models. During model development, we utilized grid search for hyperparameter tuning on select classifiers to identify optimal parameter combinations, which notably improved performance for models like AdaBoost and KNN on the internal validation set. However, most models showed poor generalization on the external validation set, with performance nearing random levels, indicating limited robustness. We also observed that models such as XGBoost and Gradient Boosting, while performing well on the training set, suffered significant AUC declines on the external validation set, suggesting overfitting. To address these challenges, we plan to employ more advanced hyperparameter optimization techniques, such as Bayesian optimization, and refine the feature extraction and selection processes in future studies to enhance model performance and clinical applicability. In conclusion, while recognizing the current limitations, we remain committed to improving model tuning and predictive outcomes in subsequent research.

## 5 Conclusion

This study investigated the relationship between sarcopenia and lung cancer prognosis, focusing on the role of the erector spinae muscles. We found that a reduction in erector spinae muscle mass was significantly associated with poor prognosis in NSCLC. Unlike traditional studies that focus on abdominal muscles, we suggest that the erector spinae may be a more relevant and important muscle group in prognostic evaluation of lung cancer. Our findings indicate that erector spinae muscle mass can serve as a valuable marker for prognosis in lung cancer patients, assisting clinicians in making treatment decisions. However, there are limitations in this study, such as a small sample size and potential reliability issues arising from the accuracy of the automatic segmentation tool. Future research should explore comprehensive assessments of different muscle groups. Furthermore, the molecular mechanisms of muscle loss in lung cancer progression should be investigated. Additionally, muscle mass assessment tools should be considered for integration into clinical practice to guide treatment decisions and improve patients’ quality of life. In conclusion, our study is the first to combine erector spinae imaging features with lung cancer research. Additionally, we developed a stable and well-performing model to predict the prognosis of NSCLC patients. This study provides new directions for the application of radiomics in cancer research.

## Data Availability

The raw data supporting the conclusions of this article will be made available by the authors, without undue reservation.
