# Supplementary material for: Study on postoperative survival prediction model for non-small cell lung cancer: application of radiomics technology workflow based on multi-organ imaging features and various machine learning algorithms
Source: Front Med (Lausanne). 2025 Feb 5;12:1517765. doi: 10.3389/fmed.2025.1517765 (PMC11835680; doi:10.3389/fmed.2025.1517765)
Supplement: Supplementary file 2 [file Data_Sheet_2.pdf]

METRICS Tool v1.0

Please fill out all conditions first for relevant sections and then all active items to calculate METRICS score.

Please note that default option is "No".

? Stands for explanation of items and conditions.

C Stands for conditional items or sections.

Items/Conditions	Definitions	Weights	Options
**Study Design**			
Item#1	? Adherence to radiomics and/or machine learning-specific checklists or guidelines	0.0368	◉ Yes ○ No
Item#2	? Eligibility criteria that describe a representative study population	0.0735	◉ Yes ○ No
Item#3	? High-quality reference standard with a clear definition	0.0919	◉ Yes ○ No
**Imaging Data**			
Item#4	? Multi-center	0.0438	◉ Yes ○ No
Item#5	? Clinical translatability of the imaging data source for radiomics analysis	0.0292	◉ Yes ○ No
Item#6	? Imaging protocol with acquisition parameters	0.0438	◉ Yes ○ No
Item#7	? The interval between imaging used and reference standard	0.0292	◉ Yes ○ No
**Segmentation** C			
Condition#1	? Does the study include segmentation?		◉ Yes ○ No
Condition#2	? Does the study include fully automated segmentation?		◉ Yes ○ No
Item#8	? Transparent description of segmentation methodology	0.0337	◉ Yes ○ No
Item#9	? Formal evaluation of fully automated segmentation C	0.0225	○ Yes ◉ No
Item#10	? Test set segmentation masks produced by a single reader or automated tool	0.0112	◉ Yes ○ No
**Image Processing and Feature Extraction**			
Condition#3	? Does the study include hand-crafted feature extraction?		◉ Yes ○ No
Item#11	? Appropriate use of image preprocessing techniques with transparent description	0.0622	◉ Yes ○ No
Item#12	? Use of standardized feature extraction software C	0.0311	◉ Yes ○ No
Item#13	? Transparent reporting of feature extraction parameters, otherwise providing a default configuration statement	0.0415	◉ Yes ○ No
**Feature Processing**			
Condition#4	? Does the study include tabular data?		◉ Yes ○ No
Condition#5	? Does the study include end-to-end deep learning?		○ Yes ◉ No
Item#14	? Removal of non-robust features C	0.0200	◉ Yes ○ No
Item#15	? Removal of redundant features C	0.0200	◉ Yes ○ No
Item#16	? Appropriateness of dimensionality compared to data size C	0.0300	◉ Yes ○ No
Item#17	? Robustness assessment of end-to-end deep learning pipelines C	0.0200	○ Yes ○ No
**Preparation for Modeling**			
Item#18	? Proper data partitioning process	0.0599	◉ Yes ○ No
Item#19	? Handling of confounding factors	0.0300	◉ Yes ○ No
**Metrics and Comparison**			
Item#20	? Use of appropriate performance evaluation metrics for task	0.0352	◉ Yes ○ No
Item#21	? Consideration of uncertainty	0.0234	◉ Yes ○ No
Item#22	? Calibration assessment	0.0176	◉ Yes ○ No
Item#23	? Use of uni-parametric imaging or proof of its inferiority	0.0117	○ Yes ◉ No

Item#24	? Comparison with a non-radiomic approach or proof of added clinical value	0.0293	(•) Yes	( ) No
Item#25	? Comparison with simple or classical statistical models	0.0176	(•) Yes	( ) No
**Testing**				
Item#26	? Internal testing	0.0375	(•) Yes	( ) No
Item#27	? External testing	0.0749	(•) Yes	( ) No
**Open Science**				
Item#28	? Data availability	0.0075	( ) Yes	(•) No
Item#29	? Code availability	0.0075	( ) Yes	(•) No
Item#30	? Model availability	0.0075	(•) Yes	( ) No
		**Total METRICS score:**	**95.0%**	
		? **Quality category:**	**Excellent**	
		? **Publication ID:**		

If you publish any work which uses this tool, please cite the following publication:

Kocak B, Akinci D'Antonoli T, Mercaldo N, et al. METhodological RadiomICs Score (METRICS): a quality scoring tool for radiomics research endorsed by EuSoMII. Insights Imaging. 2024;15(1):8. Published 2024 Jan 17. doi:10.1186/s13244-023-01572-w
