# Supplementary material for: Study on postoperative survival prediction model for non-small cell lung cancer: application of radiomics technology workflow based on multi-organ imaging features and various machine learning algorithms
Source: Front Med (Lausanne). 2025 Feb 5;12:1517765. doi: 10.3389/fmed.2025.1517765 (PMC11835680; doi:10.3389/fmed.2025.1517765)
Supplement: Supplementary file 3 [file Supplementary_file_1.docx]

**Inclusion Criteria:**

1. Age >18 years, no gender restriction.
2. Clear surgical indication and ECOG performance status 0-1.
3. No prior immunotherapy or chemotherapy for metastatic lesions.
4. Judged by clinical physicians to tolerate surgery, with functional bone marrow, liver, kidney, heart, lung, and nervous system.
5. No active autoimmune disease, active hepatitis B, or HIV infection; no need for systemic corticosteroids (over 10 mg/day prednisone) or other immunosuppressive drugs during treatment.
6. Underwent surgical treatment in our hospital with postoperative pathology indicating lung adenocarcinoma.
7. Voluntarily participate and cooperate with the research, including treatment and follow-up, data collection by researchers, avoiding other treatments, and signing informed consent.

**Exclusion Criteria:**

1. Surgical contraindications or distant metastasis at diagnosis (e.g., brain, bone, liver).
2. Poor cardiopulmonary function, unable to tolerate surgery.
3. History of other malignancies.
4. Require surgical treatment for myocardial infarction, cerebral infarction, or other thromboembolic diseases.
5. Severe infection.
6. Postoperative pathology indicating other types of lung cancer, such as squamous cell carcinoma or small cell lung cancer.
7. Pregnant or breastfeeding women, or those planning to conceive.
8. Received other anti-tumor traditional Chinese medicine treatment, chemotherapy, or physical therapy within the past 4 weeks.
9. Severe mental illness or other reasons deemed unsuitable for the study by the researcher.
